# Supplementary material for: Registered report: How open do you want your science? An international investigation into knowledge and attitudes of psychology students
Source: PLoS One. 2022 Feb 28;17(2):e0261260. doi: 10.1371/journal.pone.0261260 (PMC8884512; doi:10.1371/journal.pone.0261260)
Supplement: Attachment — Submitted filename: Reviewer replies.docx [file pone.0261260.s001.docx]

Dear Dr Soh, dear reviewers,

Thank you very much for the swift turnaround on our registered report protocol entitled “Registered Report: How Open Do You Want Your Science? An International Investigation into Knowledge and Attitudes of Psychology Students”.

My co-authors and I are delighted to see that only minor revisions are necessary for publication. Please find below our responses to the feedback.

**Editorial aspects:**

1. *Please ensure that your manuscript meets PLOS ONE's style requirements, including those for file naming. The PLOS ONE style templates can be found at*
   [*https://journals.plos.org/plosone/s/file?id=wjVg/PLOSOne_formatting_sample_main_body.pdf*](about:blank) *and* 
   [*https://journals.plos.org/plosone/s/file?id=ba62/PLOSOne_formatting_sample_title_authors_affiliations.pdf*](about:blank)

We have made the necessary edits to our manuscripts, renamed files where applicable, and used the PACE tool to adapt our figures to journal standards. Please not that purely stylistic edits are not included in the tracked changes version of the manuscript, but only the finalised version.

*2. Your ethics statement should only appear in the Methods section of your manuscript. If your ethics statement is written in any section besides the Methods, please move it to the Methods section and delete it from any other section. Please ensure that your ethics statement is included in your manuscript, as the ethics statement entered into the online submission form will not be published alongside your manuscript.*

Thank you for the clarification. The ethics statement now only appears in the methods section of the protocol.

**Replies to Reviewer #1*:***

*Overall, the protocol is well planned and written. There are only a couple of small issues I would like to point out: the colour scale in Figure 5 makes it difficult to distinguish differences, and the last sentence on page 14 is missing a closing parenthesis.*

Thank you very much for your feedback, we are glad to see that our protocol is received as well structured, and the content is clear to people outside the team.

We have fixed the issue with the parenthesis on page 14.

We would like to thank you for your comment on Figure 5. We would like to clarify that the gradient colour is only an addition, which we hoped would make it more intuitive to read, but not technically necessary. As the differences in colour strength are determined by the data, there is, unfortunately, nothing we can do to distinguish the lines more without misrepresenting the data. Nevertheless, we hope that the graphic, together with the in-text explanation sufficiently explain this aspect of the sample size estimation.

**Replies to Reviewer #2*:***

*the study is well designed and prepared, but the main issue is the English Language that is used especially the tenses; major parts of the study talks about the future and the study is already written and has been sent for publication. I think it is better to use past tense and present perfect as required in each place, or to give the study a language expert to review it.*

Thank you very much for your feedback and review, we are glad to learn that you find our study design in the registered report protocol well designed and prepared.

As for the tenses used throughout the manuscript, we have re-checked the writing with native speakers an believe they are used correctly. As this is a protocol, most sections use future tense(s), as they describe the study plan to be carried out post-protocol publication. Only the sections on sample size estimation and the section on insights from the pilot study are written in past tense, as they refer to analyses and simulations already conducted in preparation for the main study.

*I also recommend adding some idea in the topic about developing a tool for assessing attitudes about open science.*

The development of the tool for assessing attitudes is first described in lines 114-116 and then quantified through research questions 1, 3, and 4. We understand that there are certainly more aspects regarding the attitudes towards Open Science which would be interesting to study, but since our questionnaire is already rather long, we would prefer not to add any further items. This would also require a recalculation of all aspects with regards to power and we would have to re-run the pilot study. As such, we hope you can understand that we would like to avoid such big changes in the research design at this point.
